# Colorectal Cancer Genetic Heterogeneity Delineated by Multi-Region Sequencing

**DOI:** 10.1371/journal.pone.0152673

**Published:** 2016-03-29

**Authors:** You-Wang Lu, Hui-Feng Zhang, Rui Liang, Zhen-Rong Xie, Hua-You Luo, Yu-Jian Zeng, Yu Xu, La-Mei Wang, Xiang-Yang Kong, Kun-Hua Wang

**Affiliations:** 1 Faculty of Environmental Science and Engineering, Kunming University of Science and Technology, Kunming, Yunnan, P.R. China; 2 Faculty of medical, Kunming University of Science and Technology, Kunming, Yunnan, P.R. China; 3 Department of Pharmacy, The First People's Hospital of Yunnan Province, Kunming, Yunnan, P.R. China; 4 Yunnan Institute of Digestive Disease, The First Affiliated Hospital of Kunming Medical University, Kunming, Yunnan, P.R. China; 5 Department of Gastrointestinal and Hernia surgery, The First Affiliated Hospital of Kunming Medical University, Kunming, Yunnan, P.R. China; 6 Jie-Shou Li Academician Workstation, Kunming, Yunnan, P.R. China; 7 Department of Pathology, First People's Hospital of Yunnan Province, Kunming, Yunnan, P.R. China; Sapporo Medical University, JAPAN

## Abstract

Intratumor heterogeneity (ITH) leads to an underestimation of the mutational landscape portrayed by a single needle biopsy and consequently affects treatment precision. The extent of colorectal cancer (CRC) genetic ITH is not well understood in Chinese patients. Thus, we conducted deep sequencing by using the OncoGxOne^™^ Plus panel, targeting 333 cancer-specific genes in multi-region biopsies of primary and liver metastatic tumors from three Chinese CRC patients. We determined that the extent of ITH varied among the three cases. On average, 65% of all the mutations detected were common within individual tumors. *KMT2C* aberrations and the *NCOR1* mutation were the only ubiquitous events. Subsequent phylogenetic analysis showed that the tumors evolved in a branched manner. Comparison of the primary and metastatic tumors revealed that *PPP2R1A* (E370X), *SETD2* (I1608V), *SMAD4* (G382T), and *AR* splicing site mutations may be specific to liver metastatic cancer. These mutations might contribute to the initiation and progression of distant metastasis. Collectively, our analysis identified a substantial level of genetic ITH in CRC, which should be considered for personalized therapeutic strategies.

## Introduction

Colorectal cancer (CRC) is one of the leading causes of death worldwide, accounting for an estimated 1.2 million new cases and 600,000 deaths every year [1]. CRC is the third most prevalent cancer and the fifth leading cause of cancer deaths in China. The incidence of CRC has also increased in the Chinese population in recent years [2]. Although considerable progress has been made regarding CRC treatment, including targeted therapy, the five-year relative survival of patients with distant metastases is only 12.5% [3]. Therefore, an extensive understanding of the molecular biology of CRC is highly desirable. These new insights would subsequently improve the treatment of CRC.

Intratumor heterogeneity (ITH) leads to an underestimation of the mutational landscape portrayed by a single needle biopsy and consequently affects treatment precision. With the development of next-generation sequencing (NGS), ITH has recently been elucidated in substantial detail in several cancer types [4–7], including CRC [8, 9]. Kim et al. [8] conducted multi-region biopsies of primary and liver metastatic regions from five CRCs through whole-exome sequencing and observed that only 19.8%–53.9% of the mutations in a given sample were universal. They also identified *APC*, *KRAS*, and *TP53* mutations in all regional biopsies. The sequencing coverage of their study is relative low, which may lead to overlook the low frequency mutation. Whereas in Kogita study, [9] they only targeted 50 cancer genes, and hence the identified the extent of CRC genetic ITH was limited. In addition the extent of CRC genetic ITH has not been established in Chinese patients.

We conducted deep coverage sequencing by using the SureSelect Target Enrichment Kit and OncoGxOne^™^ Plus panel of DNA from multi-region biopsies of primary and liver metastatic tumors of three Chinese CRC patients to characterize CRC ITH in Chinese patients. We determined the mutation landscape and identified the substantial level of genetic ITH in CRC.

## Materials and Methods

### Patients and samples

Three patients with multi-regional primary colon carcinoma surgically resected in the First People’s Hospital of Yunnan Province between October 2013 and April 2014 were enrolled in the study. Pathological tumor staging was conducted in accordance with the TNM classification (Table 1). This study was approved by the ethics committee of The First People's Hospital of Yunnan Province (2014YXLH029). All patients in the study provided written informed consent for the use of resected tissue.

**Table 1:** Basic patient characteristics.

Sample	Age	Gender	Location	Cell Type	Differentiation	T stage	N stage	M stage
P1	62	male	colon	adenoca	Moderately	2	1	0
P2	60	female	colon	adenoca	Moderately	2	2	0
P3	71	female	colon	adenoca	Hight and Moderately	3	2	2

### DNA isolation

The Formalin-fixed, paraffin-embedded (FFPE) specimens were subjected to histological review, only those containing sufficient tumor cells (at least 70% tumor cells), as determined by hematoxylin and eosin staining, and matched normal tissues were selected for DNA isolation. Genomic DNA was isolated by using the QIAamp^®^ DNA Mini Kit (Qiagen, Hilden, Germany) according to the manufacturer’s procedure with slight modifications. Genomic DNA samples were quantified by using the NanoDrop 2000 spectrophotometer (Agilent, Santa Clara, CA, USA). The isolated DNA was stored at −80°C until analysis.

### Microsatellite testing

The microsatellite instability (MSI) status was determined for each case by using five mononucleotide or dinucleotide microsatellites markers (BAT25, BAT26, D17S250, D2S123, and D5S346) [10]. Primers were 5′-labeled with HEX, FAM, or TET (Sangon Biotech Co., Ltd., Shanghai, China). All microsatellite loci were amplified from matched normal and tumor DNA through fluorescence multiplex polymerase chain reaction (PCR). Then, the PCR products were sequenced on the ABI 3730XL automated sequencer by using a fragment analysis software (Gene Scan, Perkin Elmer, Waltham, MA, USA). Microsatellite marker stability was analyzed by using the GeneMapper software. The MSI status was classified as MSI-high if ≥30% of markers were unstable, MSI-low if <30% of markers were unstable, and no shifts or additional peaks for microsatellite stable (MSS) colon cancer.

### Target enrichment and sequencing

Sample sequence enrichment and library preparation was conducted by using the SureSelect Target Enrichment Kit (Agilent, Santa Clara, CA, USA) and OncoGxOne^™^ Plus cancer panel (GENEWIZ, Inc., South Plainfield, NJ, USA) according to the manufacturer’s instructions. OncoGxOne Plus, a targeted oncology panel, contains 333 cancer-specific genes (including all known cancer driver genes and 64 targeted and chemotherapy related genes). Briefly, 200 ng of genomic DNA from each sample was fragmented by acoustic shearing on a Covaris instrument. Fractions of 150–200 bp were ligated to SureSelect Adaptor Oligo and PCR-amplified for 10 cycles. For target enrichment, the entire library was hybridized by using the SureSelect Oligo Capture Kit for 16 or 24 h at 65°C. Biotinylated hybrids were captured, and the enriched libraries were completed under 16 PCR cycles. The resulting purified libraries were pooled and sequenced by using the Illumina^®^ MiSeq^™^ NGS instrument for 2 × 150 paired-end sequencing reads (Illumina, San Diego, CA, USA) according to the manufacturer’s protocols. The genes in the OncoGxOne^™^ Plus cancer panel are listed in S1 Table.

### Bioinformatics analysis

Sequencing data were accessed through the MiSeq Reporter. Data quality was checked by using FastQC (http://www.bioinformatics.bbsrc.ac.uk/projects/fastqc/) and then aligned to the human reference genome (hg19) by using the Burrows–Wheeler alignment tool to generate a bam file [11]. Variant calling was conducted by using the Genome Analysis Toolkit, by default [12]. Raw variant calls were filtered out by using allele frequencies > 5% and altered reads>7X. Then, the resulting variants were annotated by the Illumina Variant Studio version 2.2.3 (Illumina, San Diego, CA, USA) and ANNOVAR [13]. Known single-nucleotide polymorphisms were excluded by using variants inthedbSNP 137 (hg19) [14] and SNPs presented in the 1000 Genomes data. The potential germinal mutations were sequenced in matched normal tissues.

### Phylogenetic analysis

We inferred ancestral relationships between primary tumors and metastases of Patient 3 by mutation profiles. Neighbor trees were constructed as previously described by Kim et al. [8], with some modifications. We used phytools [15] to calculate neighbor distances and neighbor algorithm implemented in the PHYLIP [16] software package to infer the phylogenetic tree.

## Results

### Overview of the NGS data

Three CRC patients (P1, P2, and P3) were included in this study. Then, 10 samples from 3 patients were sequenced, with a median coverage of 250 reads. We identified ~8,000 somatic single-nucleotide variations (SNVs) (4,444 to 8,348) and ~850 (457 to 1,154) somatic insertions and deletions (indels) in the 10 biopsy samples from the 3 CRC cases (Table 2).

**Table 2:** Summary of somatic SNVs and small indels.

Category	P1-1 ^b^	P1-2	P2-1	P2-2	P2-3	P3-1	P3-2	P3-3	L1	L2
**A:SNV**										
downstream	31	46	47	49	45	31	36	31	30	33
intergenic	1258	4046	1334	2054	1314	973	2181	1648	922	4169
intronic	1728	2137	2217	2932	2483	1592	1656	1562	1656	1701
Within_non_coding_gene	596	725	606	674	622	502	533	480	489	543
upstream	28	51	32	54	41	38	35	24	53	55
upstream;downstream	24	38	22	20	25	19	24	18	18	24
UTR3	522	579	632	740	678	559	571	555	594	590
UTR5	104	128	116	125	122	125	124	123	129	124
stop_gained^a^	7	7	5	4	5	4	5	6	4	8
nonsynonymous_CODING^a^	262	271	238	244	241	257	265	250	258	264
synonymous_CODING	308	316	284	292	287	285	288	282	286	291
splicing^a^	4	4	2	2	2	4	4	4	5	5
**B:indel**										
downstream	6	11	9	12	9	10	10	9	7	8
intergenic	58	177	107	124	101	69	100	72	62	135
intronic	226	530	532	632	541	366	391	341	305	316
Within_non_coding_gene	32	60	56	64	60	52	53	45	52	63
upstream	3	10	6	10	6	5	7	4	6	8
UTR3	107	248	240	263	236	216	240	209	193	203
UTR5	11	28	26	31	31	28	29	26	31	31
frameshift_coding^a^	10	11	12	13	13	14	13	12	12	11
nonframeshift_coding^a^	4	4	4	4	4	3	3	3	3	3
splicing^a^			1	1	1	1	1	1	1	1

^a^ Mutations from these categories might alter protein.

^b^ P1-1 and P1-2 are primary-tumor regions of colectomy specimen from patient 1; P2-1~P2-3 are primary-tumor regions of colectomy specimen from patient 2; P3-1~P3-3 are primary-tumor regions of colectomy specimen from patient 3, L1 and L2 are two regions of the excised liver metastasis lesion from patient 3.

### Patient 1

A 62-year-old male was diagnosed with MSS colon cancer, with a 3.5 cm × 3 cm × 1.2 cm tumor. Two regions (P1-1 and P1-2) of the primary tumor exhibited a moderately differentiated adenocarcinoma, and the pathological stage of the tumor was T2N1M0. The patient received no prior treatment.

**Fig 1:**
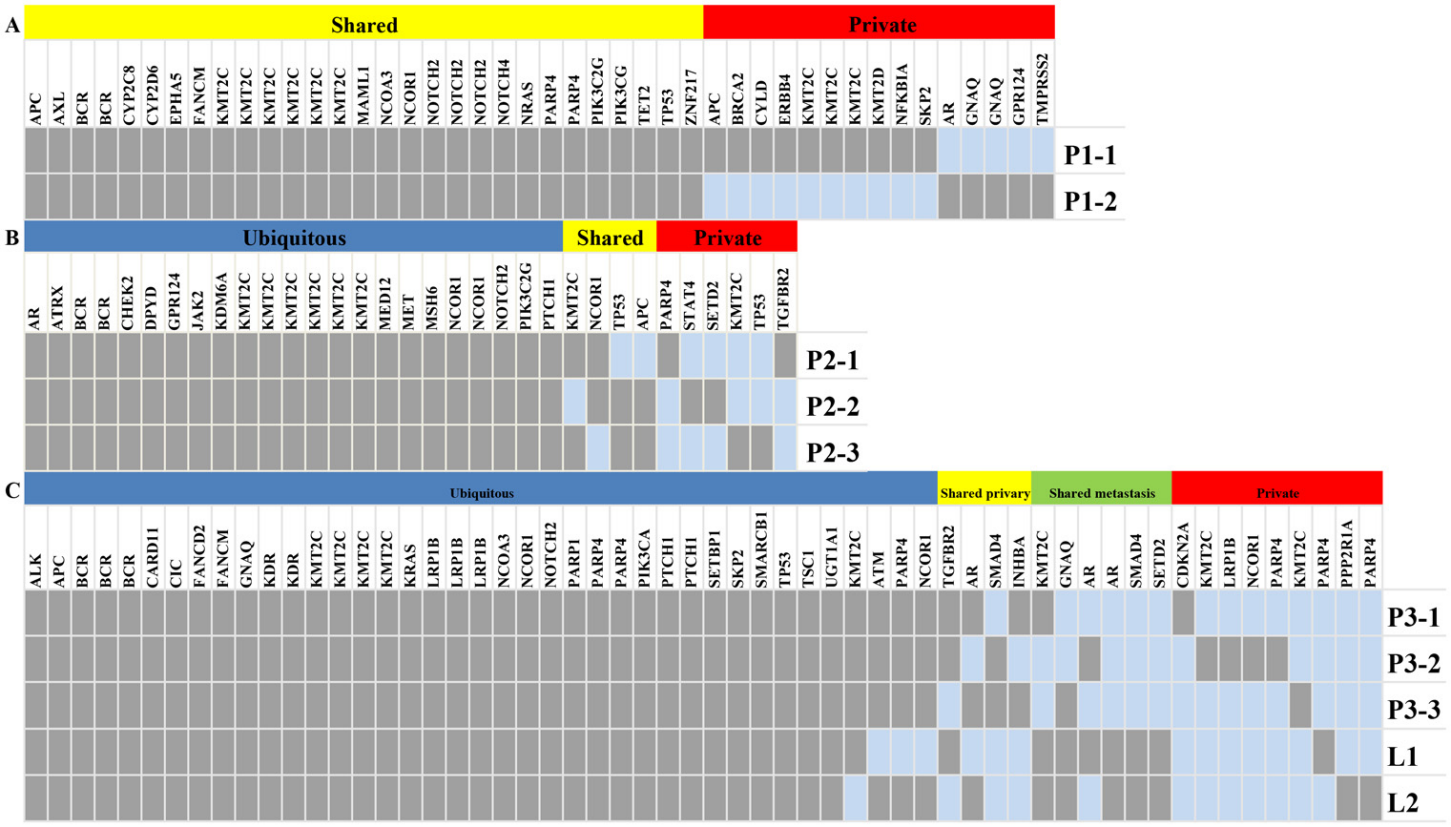
Genetic ITH in three Patient. **A Genetic ITH in Patient 1.** The regional distribution of 45 somatic variants in 3 primary tumor regions (P1-1 and P1-2); **B Genetic ITH in Patient 2.** The regional distribution of 33 somatic variants in 3 primary tumor regions (P2-1, P2-2, and P2-3); **C Genetic ITH in Patient 3.** The regional distribution of 58 somatic variants in 3 primary and 2 metastatic regions. The heat map indicates the presence (gray) or absence (dark blue) of a mutation in each region. The color bars above the heat map specify the categories of mutations.

The nonsynonymous somatic point mutations and indels that lead to protein alterations are summarized in S2 Table. Their distributions in different regions across the tumor were mapped (Fig 1A). By comparison of the mutation spectra between primary regions, we determined that 24% (11/45) of the mutations were specific to P1-1, whereas 13% (6/45) of the mutations were specific to P1-2. The remaining 63% of the mutations were shared by both regions. By comparing our result with the 127 significantly mutated genes identified by Kandoth et al. [17], we determined that P1-1 and P1-2 contained *APC* (428_429del), *KIT* (A755T), *KMT2C* (R909K, C391X, G315S D348N, P309S, and .Y816_I817delinsX), *NCOR1* (S63L), *NRAS* (G12D), and *PIK3CG* (K344Q) mutations. P1-1 contained seven additional mutations, namely, *APC* (Q706X), *KMT2D* (3860_3861del), *KMT2C* (S338L, K306fs, and a splicing site mutation) *BRCA2* (T3030fs), and *ERBB4* splicing site mutation. P1-2 contained another *AR* (57_58del) mutation.

### Patient 2

A 60-year-old female was diagnosed with MSS colon cancer, with a 3.8 cm × 3.5 cm × 1.2 cm tumor. Three regions (P2-1, P2-2, and P2-3) of the primary tumor exhibited a moderately differentiated histology and right hepatic lobe metastasis. Multiple lymph node metastases (pN2) were detected, and the pathological stage of the tumor was T2N4M0. The patient received no prior treatment.

For Patient 2, targeted resequencing of DNA from the three regions of primary tumor was conducted. This process revealed 25 nonsynonymous point mutations and 8 indels (S2 Table). Their distributions in different regions across the tumor were mapped (Fig 1B) as described by Gerlinger et al. [5]. We distinguished the 33 mutations into 23 ubiquitous mutations (those that presented in all regions in each CRC), 6 private mutations (those that presented in only one region), and the rest called shared mutations. On average, a single biopsy exhibited 28 somatic mutations, accounting for 85% of all the mutations observed in this tumor. The mutation landscapes of the different regions were highly similar to one another.

We identified several significantly mutated genes, including *ATRX* (P571A), *CHEK2* (P358S), *KDM6A* (A1038T), *NCOR1* (N208K, S63L), *KMT2C* (A746S, G892R, P309S, R909K, D348N, and Y816_I817delinsX), and *AR* (57_59del), which were ubiquitous mutations. The *TGFBR2* (E125fs) was specific to P2-1, *SETD2* (I1608V) mutation was specific to P2-2, *TP53* (T218P), *KMT2C* (K339N) was specific to P2-3. *APC* (L693fs) and TP53 (R89W) was shared by P2-2 and P2-3. *NCOR1* (K85N) was shared by P2-2 and P2-1. *KMT2C* (Q755X) is shared by P2-1 and P2-3.

### Patient 3

A 71-year-old female was diagnosed with MSS-high and moderately differentiated colon cancer, with a 9 cm × 5 cm × 3 cm primary tumor and a 1.5 cm × 1 cm × 0.5 cm right hepatic lobe metastasis. Three regions (P3-1, P3-2, and P3-3) of the primary cancer and two metastatic regions were resected, and the pathological stage of the tumor was T3N2M2. The patient received no prior treatment. For this patient, targeted resequencing was conducted on the DNA from the three regions (P3-1, P3-2, and P3-3) of the primary tumor and two metastatic regions. The process revealed 47 nonsynonymous point mutations and 11 indels (S2 Table). Their distributions in different regions across the tumor were mapped (Fig 1C).

**Fig 2:**
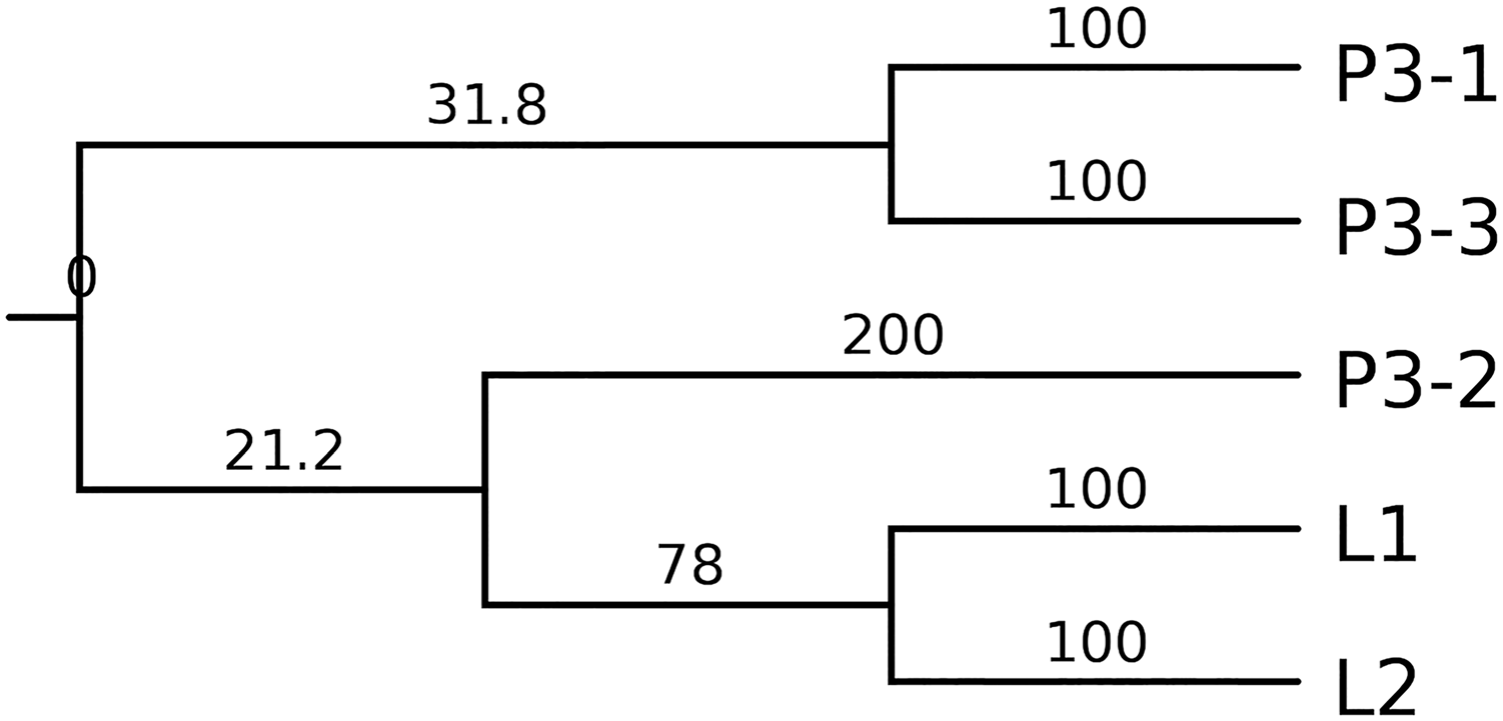
Phylogenetic relationship between multi-regional biopsies inferred from somatic mutations. Branch lengths in the tree are proportional to the number of nonsynonymous mutations in the corresponding branches.

We constructed a phylogenetic tree (Fig 2), which showed a branched, rather than a linear, tumor evolution, to represent the evolutionary history of the CRC. P3-2 mutation spectra were more similar to L1 and L2, indicating that the origins of distant metastases could be traced to one of the primary sites.

Overall, target sequencing identified 45 somatic mutations per biopsy, accounting for 78% of all mutations observed in this tumor. 60% of all the mutations detected by multi-region sequencing in the colectomy specimen were ubiquitous mutations present in all regions. When we analyzed the primary cancer, an average of 45 somatic mutations were detected per biopsy, which accounted for 87% of all the mutations identified in this tumor. For the metastatic sites, a single biopsy revealed 92% of all the mutations detected in this tumor.

In the significantly mutated genes, we determined that *KRAS* (G13D), *PIK3CA* (E545), *TP53* a splicing site mutation, *APC* (E854fs), *KMT2C* (E141G, K339N, P309S, and Y816_I817delinsX), *SETBP1* (Q1558L), and *NCOR1* (S63L) were ubiquitous mutations, whereas *CDKN2A* (D74A), *SMAD4* (W168X), *NCOR1* (N208K), *KMT2C* (G315C and K306fs) and *INHBA* (V58I) were specific to the primary cancer. *PPP2R1A* (E370X), *SETD2* (I1608V), *SMAD4* (G382T), and *AR* splicing site mutations were specific to liver metastatic cancer. However, these mutations were absent in the primary cancer regions. The *KRAS*, *TP53*, and *PIK3CA* mutations, which have often been used as molecular biomarkers in CRC, also presented in all the regions.

### Metastatic tumor-specific mutations

By comparing the mutation landscape between primary and metastatic regions, we detected that several mutations were specific to the metastatic regions. SMAD4 is a key transducer of transforming growth factor-β superfamily signaling, which regulates cell proliferation, differentiation, and apoptosis [18]. Loss of Smad4 is correlated with CRC metastasis [19–21]. Meanwhile, AR belongs to a family of nuclear receptors that act as transcription factors. AR has been shown to regulate cell migration by suppressing the nuclear factor kappa B/matrix metallopeptidase 9 pathway and further inhibiting hepatocellular carcinoma metastasis [22, 23]. AR splice variants are known to promote tumor metastasis [24]. The *PPP2R1A* gene encodes the structural subunit of the PP2A enzyme, which is a highly conserved serine/threonine phosphatase that serves a broad spectrum of biological roles, including the negative regulation of signal transduction, cell cycle progression [25], and gene expression. PPP2R1A facilitates tumor cell–lymphatic endothelial cell interactions during melanoma cell metastasis [26].

## Discussion

The multi-region mutation landscape analysis of the three CRC tumors provided substantial evidence of ITH, with the extent of ITH varying among the three cases. In Patient 1, we sequenced two spatially separated sites of primary cancer. Their mutational spectra exhibited a 63% overlap. In Patient 2, a single biopsy exhibited 28 somatic mutations on average, accounting for 85% of all the mutations observed in this tumor. By contrast, in Patient 3, 78% of all the mutations were observed in this tumor. Of all the somatic mutations revealed by multi-region sequencing, 30% to 40% were heterogeneous and thus cannot be detected in every sequenced region. Kim et al. [8] determined that 46%–80% of the mutations were undetectable across all the regional biopsies in their cohort. The level of genetic ITH in their samples was higher than that in ours. The sequencing results of the primary cancers of Patients 1 and 2 were more similar to each other than those of the samples from the aforementioned study. These findings imply that single tumor biopsies cannot accurately represent a tumor’s genomic mutational landscape, which significantly affects targeted therapy.

We constructed a phylogenetic tree by using the data from Patient 3. The tree indicated that CRC evolved in a branched, rather than a linear, manner. The branched evolution model has been validated in many types of cancers [27], including CRC [8]. Traditionally, CRC is considered to evolve in a linear manner [28]. Recent studies showed that both evolution models exist in CRC [29].

Branched tumor evolution underlines the necessity to target mutations located in the main trunk of the phylogenetic tree. We determined that *KMT2C* and *NCOR1* (S63L) were mutated in all regions compared with the significantly mutated genes reported by Kandoth et al. [17]. KMT2C is a member of the human trithorax/mixed-lineage leukemia family [30] and is involved in histone modification. *KMT2C* is frequently altered in many types of cancer, including liver cancer [31], cutaneous squamous cell carcinoma [32], and esophageal squamous cell cancer [33]. In our research, we determined that each patient has at least seven mutations in *KMT2C*, the clinical significance of which remains to be validated. We also observed a *NCOR1* missense mutation. NCOR1 is a 270 k nuclear receptor corepressor [34] that participates in ligand-dependent transcriptional repression by the estrogen receptor-α [35]. *KMT2C* and *NCOR1* may represent two potential targetable genes in the setting of heterogeneity.

*KRAS*, *TP53*, and *PIK3CA* are the most significantly mutated genes in CRC [36]. However, only Patient 3 had these mutations in our cohort. These mutations were identified in all regions obtained from the patient and showed high concordance between matched primary and metastatic CRC. This result is consistent with that of Brannon et al. [37]. The primary cancer mutation spectra accurately represent those of the metastatic lesions in terms of currently available predicted CRC biomarkers.

*PPP2R1A* (E370X), *SETD2* (I1608V), *SMAD4* (G382T), and *AR* splicing site mutations were determined to be potential metastatic tumor-specific mutations. We suppose that these mutations could drive CRC metastasis. Given the limited sample size, the frequency of these mutations in CRC metastases must be assessed in a larger cohort. Further functional studies are necessary to address their roles in CRC metastasis.

Genomic analysis from single needle biopsies may underestimate the mutational landscape of heterogeneous tumors. ITH may partly explain the poor validation of CRC biomarkers due to sampling bias. Reconstructing the tumor clonal structure and identifying the ubiquitous alterations located in the trunk of the phylogenetic tree may contribute to the discovery of more effective biomarkers and therapeutic approaches.

In conclusion, we identified the substantial extent of ITH in CRC. In the context of this phenomenon, we determined that CRC evolved in a branched manner and several molecular events may contribute to CRC progression. We investigated only three cases because of financial constraints. Further studies with large sample sizes should be conducted to confirm these findings.

## Supporting Information

S1 Table OncoGxOneTMPlus Cancer gene list. (PDF)

S2 Table Genetic alterations per patient. (XLSX)
