# Supplementary material for: Colorectal Cancer Genetic Heterogeneity Delineated by Multi-Region Sequencing
Source: PLoS One. 2016 Mar 29;11(3):e0152673. doi: 10.1371/journal.pone.0152673 (PMC4811559; doi:10.1371/journal.pone.0152673)
Supplement: S1 Table — (PDF) [file pone.0152673.s001.pdf]

OncoGxOne™Plus (333)

ABL1	CARD11	DIS3	FGF7	JAK2	MYCL	PNRC1	SOX2
ACVR1B	CASP8	DNMT1	FGFR1	JAK3	MYCN	PPP2R1A	SOX9
AKT1	CBFB	DNMT3A	FGFR2	JUN	MYD88	PRDM1	SPEN
AKT2	CBL	DOT1L	FGFR3	KAT6A	NBN	PRKAR1A	SPOP
AKT3	CCND1	DPYD	FGFR4	KDM5A	NCOA3	PRKDC	SRC
ALK	CCND2	EGFR	FLT1	KDM5C	NCOR1	PRSS8	SRSF2
ALOX12B	CCND3	ELF3	FLT3	KDM6A	NF1	PTCH1	STAG2
AMER1	CCNE1	EP300	FLT4	KDR	NF2	PTEN	STAT1
APC	CD79A	EPHA3	FOXL2	KEAP1	NFE2L2	PTPN11	STAT3
APCDD1	CD79B	EPHA5	FUBP1	KIT	NFKBIA	RAD50	STAT4
AR	CDC73	EPHB1	GATA1	KMT2A	NKX2-1	RAD51	STK11
ARAF	CDH1	EPOR	GATA2	KMT2C	NOTCH1	RAD51B	SUFU
ARFRP1	CDK12	ERBB2	GATA3	KMT2D	NOTCH2	RAD51C	SYK
ARID1A	CDK4	ERBB3	GID4	KLF4	NOTCH3	RAD51D	TBX3
ARID1B	CDK6	ERBB4	GNA11	KLHL6	NOTCH4	RAD52	TET2
ARID2	CDK8	ERG	GNA13	KRAS	NPM1	RAD54L	TGFBR2
ASXL1	CDKN1B	ESR1	GNAQ	LMO1	NRAS	RAF1	TIPARP
ATM	CDKN2A	ETV1	GNAS	LRP1B	NSD1	RARA	TMPRSS2
ATR	CDKN2B	ETV4	GPR124	MAML1	NTRK1	RB1	TNFAIP3
ATRX	CDKN2C	ETV5	GRIN2A	MAP2K1	NTRK2	REL	TNFRSF14
AURKA	CEBPA	ETV6	GSK3B	MAP2K2	NTRK3	RET	TOP1
AURKB	CHEK1	EWSR1	H3F3A	MAP2K4	NUP93	RICTOR	TP53
AXIN1	CHEK2	EZH2	HGF	MAP3K1	PAK3	RNF43	TPMT
AXL	CHUK	FAM46C	HIST1H3B	MAP3K13	PAK7	ROS1	TRAF7
B2M	CIC	FANCA	HLA-A	MAPK1	PALB2	RPA1	TRRAP
BACH1	CRBN	FANCC	HLA-B	MCL1	PARP1	RPTOR	TSC1
BAP1	CREBBP	FANCD2	HLA-C	MDM2	PARP2	RUNX1	TSC2
BARD1	CRKL	FANCE	HNF1A	MDM4	PARP3	RUNX1T1	TSHR
BCL2	CRLF2	FANCF	HRAS	MED12	PARP4	SETBP1	TYMS
BCL2L2	CSF1R	FANCG	IDH1	MEF2B	PAX5	SETD2	U2AF1
BCL6	CTCF	FANCI	IDH2	MEN1	PBRM1	SF3B1	UGT1A1
BCOR	CTNNA1	FANCL	IGF1	MET	PDGFRA	SH2B3	VHL
BCORL1	CTNNB1	FANCM	IGF1R	MITF	PDGFRB	SKP2	WISP3
BCR	CUL4A	FAT3	IGF2	MLH1	PDK1	SMAD2	WT1
BLM	CUL4B	FBXW7	IGF2R	MPL	PHF6	SMAD3	XPO1
BRAF	CUX1	FGF10	IKBKE	MRE11A	PIK3C2G	SMAD4	XRCC1
BRCA1	CYLD	FGF14	IKZF1	MSH2	PIK3C3	SMARCA4	XRCC3
BRCA2	CYP17A1	FGF19	IL7R	MSH6	PIK3CA	SMARCB1	ZNF217
BRIP1	CYP2C8	FGF23	INHBA	MTHFR	PIK3CG	SMARCD1	ZNF703
BTG1	CYP2D6	FGF3	IRF4	MTOR	PIK3R1	SMO	
BTK	DAXX	FGF4	IRS2	MUTYH	PIK3R2	SOCS1	
C11orf30	DDR2	FGF6	JAK1	MYC	PMS2	SOX10	
